# Outcomes After Resection for Multiple Intrahepatic Cholangiocarcinoma—A National Population-Based Study

**DOI:** 10.1245/s10434-025-18983-2

**Published:** 2026-01-05

**Authors:** Hannes Jansson, Helena Taflin, Bergthor Björnsson, Jozef Urdzik, Oskar Hemmingsson, Jenny Lundmark Rystedt, Stefan Gilg, Per Sandström, Ernesto Sparrelid

**Affiliations:** 1 Division of Surgery and Oncology, Department of Clinical Science, Intervention and Technology, Karolinska Institutet, Karolinska University Hospital, Stockholm, Sweden; 2 Department of Transplantation, Institute of Clinical Sciences, University of Gothenburg, Sahlgrenska Academy, Gothenburg, Sweden; 3 Department of Transplantation at Sahlgrenska University Hospital, Region Västra Götaland, Gothenburg, Sweden; 4 Department of Biomedical and Clinical Sciences, Linköping University, Linköping, Sweden; 5 Clinical Department of Surgery in Linköping, Region Östergötland, Linköping, Sweden; 6 Department of Surgical Sciences, Uppsala University, Uppsala, Sweden; 7 Department of Diagnostics and Intervention, Umeå University, Umeå, Sweden; 8 Department of Surgery, Skåne University Hospital, Lund, Sweden; 9 Department of Clinical Sciences, Lund University, Lund, Sweden

**Keywords:** Intrahepatic cholangiocarcinoma, Resection, Prognosis, Biliary tract cancer, Liver resection

## Abstract

**Background:**

Tumor multiplicity is a negative prognostic factor in intrahepatic cholangiocarcinoma (iCCA) and the role of surgical resection in multiple iCCA remains unclear.

**Patients and Methods:**

Data were extracted from the Swedish quality registry for cancers of the liver and biliary tract, for all patients undergoing surgery for iCCA (2010–2021). Validation was performed with all Swedish hepatobiliary referral centers, including a comparison cohort of patients with liver-only multiple iCCA and nonsurgical therapy. The primary endpoint was overall survival (OS).

**Results:**

Out of 338 patients operated for iCCA, 284 had resectable tumors and 54 (16.0%) unresectable disease at exploration. In the resection and exploration groups, 46 (16.2%) and 11 patients (20.4%), respectively, had multiple lesions. A majority of patients with resection for multiple iCCA had two or three lesions (63.0%), with median OS 27.1 months (95% CI 18.8–35.5), compared with 5.3 months (95% CI 3.8–6.8) for patients with unresectable disease (*P* < 0.001). For patients with four or more lesions, OS with resection was similar as with unresectable disease (*P* = 0.922). In multiple iCCA, resection was associated with better performance status (*P* = 0.007), negative lymph nodes (*P* = 0.028), and smaller tumors (*P* = 0.004). With adjustment including these factors, resection was not significantly associated with OS (*P* = 0.090).

**Conclusions:**

Resection for multiple iCCA with four or more lesions was not associated with a survival benefit compared with exploration. With selection to surgery for patients with good performance status, smaller tumors and no signs of lymph node metastasis, resection for two or three lesions yielded median OS above 2 years.

**Supplementary Information:**

The online version contains supplementary material available at 10.1245/s10434-025-18983-2.

Tumor multiplicity is a negative prognostic factor for patients with intrahepatic cholangiocarcinoma (iCCA),^1,2^ but the impact on therapeutic options and long-term outcomes remains debated.^3,4^ In studies of locoregional oncological therapy, the presence of multiple lesions has been employed as a criterion for unresectability.^5^ While previous guidelines have included multiple hepatic lesions as a contraindication to surgery,^6^ recent updates have stated that resection can be considered in selected patients.^7^ Current data on outcomes for patients with multiple iCCA mainly derive from single- and multicenter cohorts, underscoring the need for stronger evidence to guide therapy and future trials.^7^ The aim of this study was therefore to analyze population-based outcomes after resection for patients with multiple iCCA using national data from the Swedish quality registry for cancers of the liver and biliary tract.

## Patients and Methods

### Study Design

The Swedish quality registry for cancers of the liver and biliary tract (SweLiv) is a prospective national diagnosis and treatment registry with a 95% coverage rate as compared with the National Board of Health and Welfare cancer registry.^8^ The registry is linked to the Swedish National Population Registry for data on vital status. For this study, all patients undergoing surgery with a registered diagnosis of iCCA during the period January 2010 to December 2021 were screened, and patients with a confirmed diagnosis of primary iCCA were included. Clinicopathological variables included World Health Organization/Eastern Cooperative Oncology Group (WHO/ECOG) performance status at diagnosis,^9^ tumor size (diameter of largest lesion), tumor number, American Joint Committee on Cancer (AJCC)/tumor node metastasis (TNM) stage,^10^ and vital status. In collaboration between all six Swedish regional specialized hepatobiliary centers, validation data including tumor number at preoperative radiological diagnosis, surgical treatment, adjuvant therapy, postoperative diagnosis, and outcome (90 day morbidity and mortality, recurrence status, date of last follow-up) were collected through pseudonymized electronic case report forms (Research Electronic Data Capture platform [REDCap]^11^). Additionally, a comparison cohort of patients with liver-only iCCA at diagnosis (i.e., iCCA and no distant metastasis according to AJCC/TNM 8th edition^10^) and nonsurgical therapy was collected from the SweLiv registry. The period for validation data collection was 1 March to 31 May 2023. The primary outcome variable was overall survival from the date of radiological diagnosis. Secondary outcome variables were disease-free survival from the date of surgery, rate of recurrence at 6 months, and postoperative complications according to the Clavien–Dindo classification.^12^ The study was approved by the Swedish Ethical Review Authority (reference no. 2022-03763-01) with waiver of informed consent for the retrospective analysis of registry and validation data. This study was reported in accordance with the Strengthening the Reporting of Observational Studies in Epidemiology (STROBE) guidelines.^13^ The STROBE checklist is presented in Supplementary Table S1.

### Statistical Analysis

Categorical variables were presented with frequencies and percentages, continuous variables with medians and interquartile ranges (IQR). Distributions were compared with the Kruskal–Wallis test and proportions with either the chi-squared test or Fisher–Freeman–Halton Exact test, as appropriate. Survival analysis was performed by the Kaplan–Meier method with log rank test and Cox regression. Median follow-up was calculated by the reverse Kaplan–Meier method.^14^ In regression analysis, surgical resection was modeled as a time-dependent variable to account for unexposed survival time.^15^ Multiple imputation with ten imputed datasets was used for missing data in independent variables in the regression analysis. Baseline characteristics and clinicopathological variables were reported with unimputed data. A two-sided *P* value < 0.05 was considered as statistically significant. Statistical analyses were performed using IBM SPSS Statistics 29.0.2.0 and 30.0.0.0 (IBM, New York, USA).

## Results

### Baseline Characteristics

**Fig. 1:**
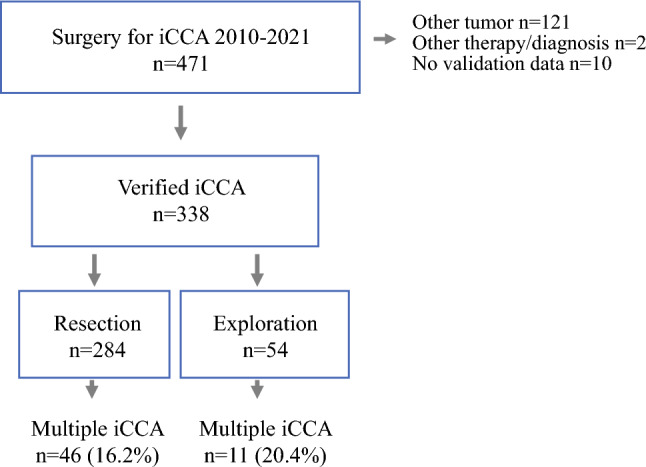
Study inclusion flowchart; *iCCA* intrahepatic cholangiocarcinoma

The study inclusion flowchart is presented in Fig. 1. A total of 471 patients, with registered surgery and a registered diagnosis of primary intrahepatic cholangiocarcinoma either pre- or postoperatively, were screened. At validation, 121 patients with resection had a different postoperative diagnosis, most frequently of perihilar cholangiocarcinoma (*n* = 80, 66.1%), and were excluded (Supplementary Table S2). One patient was excluded because of transplantation for a perihilar cholangiocarcinoma, while another was excluded because of repeat resection for recurrent iCCA. Finally, for ten patients, no clinical validation data were available.

A total of 338 patients with confirmed iCCA, 284 with resectable tumors (84.0%) and 54 (16.0%) with a finding of unresectable disease at exploration, were included. In the resection and exploration groups, 46 (16.2%) and 11 patients (20.4%), respectively, had multiple lesions. A majority of patients with resection for multiple iCCA had two or three lesions (29 patients, 63.0%). There was an association between tumor size and the number of lesions (*P* < 0.001). Baseline characteristics and clinicopathological data for patients with resection, according to number of lesions, are presented in Table 1. Most patients underwent contrast-enhanced computed tomography (CT) in combination with magnetic resonance imaging (MRI) preoperatively. Only a minority of patients underwent preoperative positron emission tomography CT (PET-CT). Anatomical resections were most frequent, with major hepatectomy performed for approximately two-thirds of patients, at similar rates for patients with one lesion and patients with multiple lesions (Table 1, *P* = 0.961). The overall R0 rate was 76.3%, with no significant difference between patients according to the number of lesions (Table 1, *P* = 0.896). The AJCC/TNM tumor (T) categories according to the 8th edition are presented in Supplementary Table S4.

**Table 1:** Clinicopathological data for patients with resection for intrahepatic cholangiocarcinoma, 2010–2021

	Missing data	1 lesion (*n* = 238)	2–3 lesions (*n* = 29)	≥ 4 lesions (*n* = 17)	*P* value
Age (years), md (IQR)	0	67 (60–73)	70 (60.5–74)	63 (50.5–71.5)	0.407^#^
Sex (female), *n* (%)	0	129 (54.2)	15 (51.7)	6 (35.3)	0.318
WHO/ECOG ≥ 2, *n* (%)	72	15 (8.5)	2 (9.5)	1 (7.1)	0.883^§^
Radiology, CT/MRI					
CT, *n* (%)	3	63 (26.8)	4 (13.8)	6 (35.3)	0.369^§^
MRI, *n* (%)		5 (2.1)	1 (3.4)	0 (0)	
CT+MRI, *n* (%)		167 (71.1)	24 (82.8)	11 (64.7)	
Radiology, PET-CT, *n* (%)	3	25 (10.6)	2 (6.9)	3 (17.6)	0.562^§^
Tumor size (largest lesion, cm), md (IQR)	36	4.5 (3.0–6.5)	6.0 (3.25–8.0)	7.0 (5.6–9.0)	**< 0.001**^#^
Resection type					
Local, *n* (%)	0	32 (13.4)	3 (10.3)	1 (5.9)	0.961^§^
Segmental, *n* (%)		24 (10.1)	2 (6.9)	1 (5.9)	
Two segments,* n* (%)		31 (13.0)	3 (10.3)	3 (17.6)	
Major (≥ 3 segments), *n* (%)		151 (63.4)	21 (72.4)	12 (70.6)	
Grade = 3, *n* (%)	64	54 (30.2)	10 (38.5)	9 (60.0)	0.054^§^
pN1, *n* (%)	0^&^	121 (50.8)	8 (27.6)	8 (47.1)	0.061
Lymph node dissection, *n* (%)	2	148 (62.7)	19 (65.5)	11 (64.7)	0.948
R1, *n* (%)	69	44 (24.6)	5 (21.7)	2 (15.4)	0.896^§^
Complications CD ≥ 3, *n* (%)	2	75 (31.8)	11 (37.9)	4 (23.5)	0.596
90-day mortality, *n* (%)	0	4 (1.7)	1 (3.4)	0 (0)	0.590^§^
Neoadjuvant therapy,* n* (%)	0	2 (0.8)	2 (6.9)	1 (5.9)	0.052^§^
Adjuvant therapy, *n* (%)	0	91 (38.2)	13 (44.8)	5 (29.4)	0.580

*P* values < 0.05 in bold type

*CD* Clavien–Dindo complication grade, *CT* computed tomography, *IQR* interquartile range, *md* median, *MRI* magnetic resonance imaging, *PET* positron emission tomography, *pN1* pathological lymph node metastasis, *R1* microscopically tumor positive resection margin, *WHO/ECOG* World Health Organization/Eastern Cooperative Oncology Group performance status

^&^N1 versus N0/NX

^§^Fisher–Freeman–Halton exact test

^#^Kruskal–Wallis test

For the comparison of surgical and nonsurgical management, patients with localized iCCA and a multidisciplinary tumor board recommendation of nonsurgical therapy were also screened (*n* = 66). At validation, 23 patients had either a different diagnosis (perihilar cholangiocarcinoma *n* = 7, gallbladder cancer *n* = 2, hepatocellular carcinoma *n* = 1), other therapy (resection *n* = 1), or no available validation data (*n* = 12). Finally, 43 patients with liver-only iCCA and nonsurgical therapy were included, 18 of whom with multiple iCCA (41.9%). When comparing clinicopathological data for patients with liver-only multiple iCCA according to treatment category (Table 2), patients with resection had better WHO/ECOG performance status (*P* = 0.007), smaller tumors (*P* = 0.004) and a lower rate of lymph node metastasis (*P* = 0.028) than patients with non-resection.

**Table 2:** Clinicopathological data for patients with liver-only multiple intrahepatic cholangiocarcinoma according to treatment category

	Missing data	Resection (*n* = 46)	Exploration (*n* = 11)	M0 non-surgical (*n* = 18)	*P* value
Age (years), md (IQR)	0	68 (56.5–73)	66 (60–71)	72.5 (61.5–76.25)	0.190^#^
Sex (female), *n* (%)	0	21 (45.7)	6 (54.5)	10 (55.6)	0.723
WHO/ECOG ≥ 2, *n* (%)	19	3 (8.6)	2 (22.2)	6 (50.0)	**0.007**^§^
Tumor number (≥ 4 lesions), *n* (%)	0	17 (37.0)	6 (54.5)	12 (66.7)	0.086
Tumor size (largest lesion, cm), md (IQR)	7	6.5 (5.0–8.3)	5.5 (3.0–9.0)	10.0 (8.0–11.7)	**0.004**^#^
Grade = 3, *n* (%)	20	19 (46.3)	2 (33.3)	6 (75.0)	0.299^§^
N1, *n* (%)	0^&^	16 (34.8)	8 (72.7)	11 (61.1)	**0.028**

*P* values < 0.05 in bold type

*IQR* interquartile range, *md* median, *N1* lymph node metastasis, *WHO/ECOG* World Health Organization/Eastern Cooperative Oncology Group performance status ^&^N1 versus N0/NX

^§^Fisher–Freeman–Halton exact test

^#^Kruskal–Wallis test

### Survival analysis

**Fig. 2:**
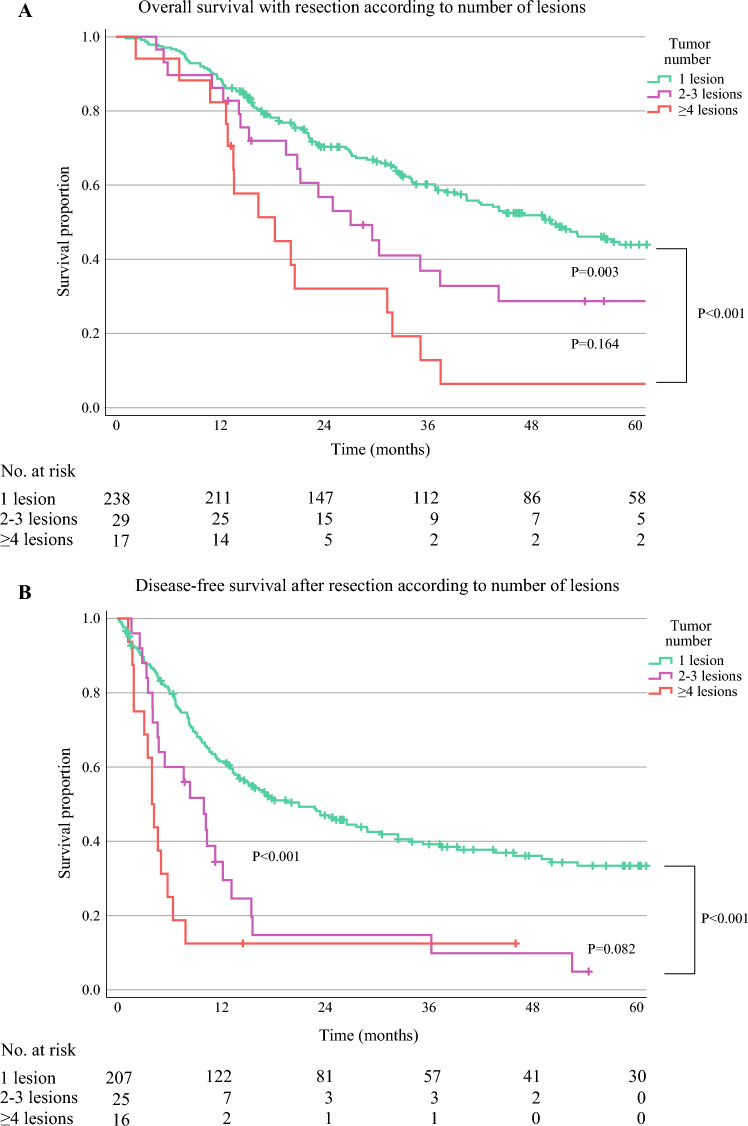
Overall (**A**) and disease-free survival (**B**) for patients with resection for intrahepatic cholangiocarcinoma, according to the number of tumor lesions; *P* by log rank test (1 versus 2–3 lesions; 2–3 versus ≥ 4 lesions; 1 versus ≥ 4 lesions)

**Fig. 3:**
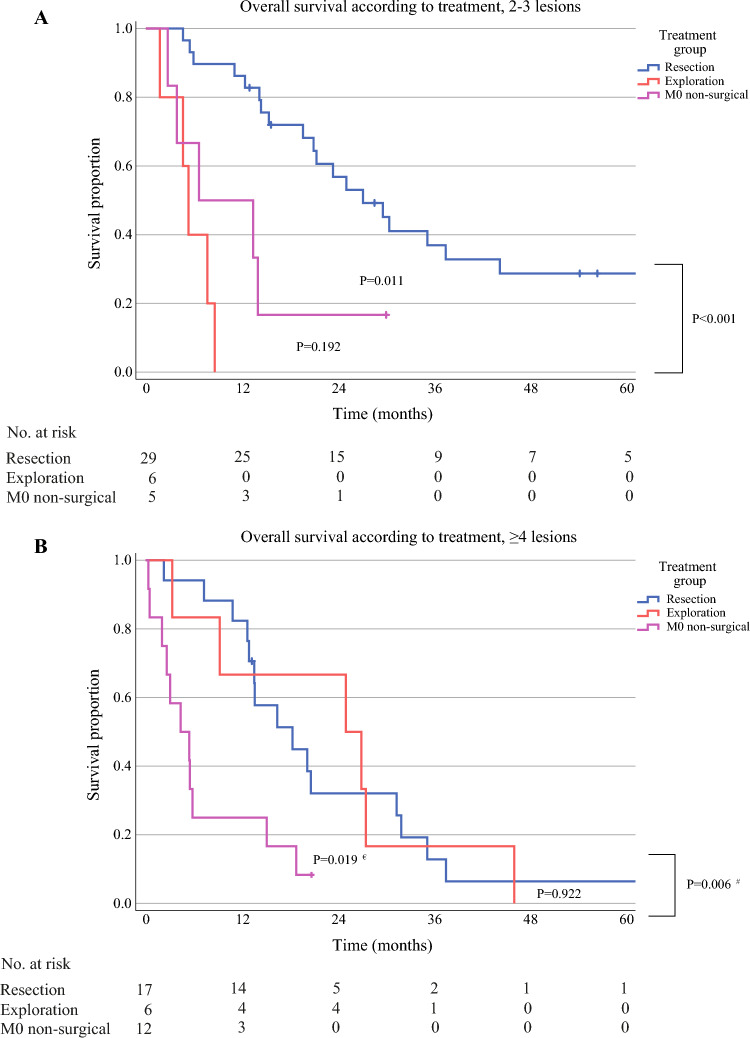
Overall survival from date of diagnosis for patients with multiple intrahepatic cholangiocarcinoma (**A**: 2 or 3 lesions; **B**: 4 or more lesions), according to treatment group; M0, no distant metastasis; *P* by log rank test (resection versus M0 nonsurgical; M0 nonsurgical versus exploration; resection versus exploration); ^#^resection versus M0 nonsurgical, ^€^exploration versus M0 nonsurgical

The median follow-up time was 63.2 months [95% confidence interval (CI) 57.1–69.4 months], with a median OS for all patients undergoing resection of 42.0 months (95% CI 33.1–50.8 months). Tumor multiplicity was negatively associated with OS (Fig. 2A, P < 0.001). For patients with resection for multiple iCCA with two or three lesions, median OS was 27.1 months (95% CI 18.8–35.5 months), compared with 5.3 months (95% CI 3.8–6.8 months) for patients with unresectable disease (Fig. 3A, *P* < 0.001). For patients with four or more lesions, OS with resection (18.4 months, 95% CI 9.4–27.3 months) was similar as for patients with unresectable disease at exploration (Fig. 3B, *P* = 0.922). For patients with resection for single iCCA, median OS was 50.4 months (95% CI 40.3–60.5 months), while for patients with resection for multiple iCCA the median OS time was 23.4 months (95% CI 16.2–30.6 months). Within the multiple iCCA groups analyzed, OS according to number was similar (two or three lesions *P* = 0.817, four or more lesions *P* = 0.312).

Resection for multiple iCCA was associated with a shorter DFS compared with resection for iCCA with one single lesion (Fig. 2B, *P* < 0.001). For patients with two or three lesions, median DFS was 10.0 months (95% CI 6.1–13.9 months), while for patients with four or more lesions, median DFS time was only 4.0 months (95% CI 3.1–5.0 months, *P* = 0.082). The rate of recurrence within 6 months was 40.0% (*n* = 10) for patients with two or three lesions and 75.0% (*n* = 12) for patients with four or more lesions (*P* = 0.028).

In the univariable Cox regression analysis of prognostic factors for OS in patients with multiple iCCA, resection (*P* = 0.025) was a positive prognostic factor, while tumor size was a significant negative prognostic factor (*P* = 0.027) (Supplementary Table S3). To assess the prognostic value of multiple lesions in relation to tumor size, subgroup analyses of OS were performed for patients with tumors up to 5 cm and for patients with tumors larger than 5 cm (Supplementary Fig. S1). In both size groups, the number of lesions was associated with OS (*P* = 0.031 and *P* = 0.008). To account for background differences between patients with and without resection, a multivariable Cox regression analysis including age, WHO/ECOG performance status, tumor size, tumor number, and lymph node metastasis was performed. In the adjusted model, resection was not significantly associated with OS (*P* = 0.090) (Supplementary Table S3).

## Discussion

In this national analysis of population-based data for patients with iCCA, resection for multiple iCCA with two or three lesions resulted in a median OS time of 27.1 months and 5-year OS above 20%. In contrast, for patients with four or more lesions, resection was not associated with a survival benefit compared with surgical exploration without resection. Patients with multiple iCCA selected for resection surgery had smaller tumors, better performance status, and a lower rate of lymph node metastasis compared with patients with no resection. The rate of postoperative complications after resection for multiple iCCA was similar to that for patients with a single lesion, as was the proportion of patients that could start adjuvant oncological therapy. With most of the study period preceding the 2019 BILCAP trial,^16^ and the subsequent introduction of adjuvant capecitabine as a standard therapy in Sweden, only less than 40% of all operated patients received adjuvant treatment. Notably, resection for multiple iCCA was associated with shorter DFS, so that patients with two or three lesions had a median DFS of 10 months, while patients with four or more lesions had a median DFS of only 4 months. During this study period, neoadjuvant therapy was rarely used in iCCA, at a rate of 1.8% for resected patients overall, and 6.5% for patients with multiple lesions.

While tumor multiplicity was strongly associated with larger tumor size as a negative prognostic factor, most patients with resection for multiple lesions had no lymph node metastasis. This likely reflects selection to surgery in multiple iCCA for patients with no radiological signs of lymph node metastasis at the preoperative evaluation. Around two-thirds of patients underwent a lymphadenectomy, with similar rates regardless of tumor number. With regard to tumor differentiation, a majority of patients with four or more lesions had high-grade tumors on postoperative histopathology, which was twice the rate to that of patients with a single lesion; although with no statistically significant difference between patients according to number. The clinical routine in Sweden during this period has been to recommend resection without preoperative biopsy at clinical diagnosis of resectable iCCA, which means that data on histological or genetic tumor characteristics are not known at the time of diagnosis for surgical candidates. Tumor size remained a negative prognostic factor on multivariable analysis. However, within subgroups according to size, tumor number was still prognostic for OS, illustrating that size was not the only driver of poor prognosis. Recently, the tumor burden score has been suggested as one way to incorporate both size and tumor number to assess prognosis in iCCA.^17^

The median OS time of 23.4 months for all resected patients with multiple iCCA seen in this national population-based study can be compared with that reported from an international multicenter cohort by Franssen et al. of 18.9 months.^5^ The proportion of patients with multiple iCCA and four or more lesions was higher in the present study (37%) compared with the study from Franssen et al. (24%).^5^ Importantly, with a direct comparison with exploration, the current study identified a survival benefit with resection only for patients with less than four lesions. For a single-center patient cohort receiving locoregional chemotherapy by hepatic arterial infusion, reported in the previously mentioned study, the proportion of patients with four or more lesions was 67% and the median OS was 20.3 months.^5^ Previous studies presenting single-center outcomes after resection for multiple iCCA have reported median OS times between 20 and 27 months, with rates of lymph node metastasis ranging from 26 to 38%.^18–21^ The present study shows that such outcomes can be reproduced in an unselected national setting as well.

This study underscores that iCCA is a rare cancer in Sweden. As a majority of patients with iCCA are diagnosed with distant metastasis at presentation,^22^ only a limited number of patients each year will be evaluated for surgery or other locoregional treatments. This should emphasize the importance of a multidisciplinary evaluation at a regional expert center, and prompt collaborative national and international prospective studies. With promising results reported for locoregional therapies, such as hepatic arterial infusion chemotherapy,^5^ and for targeted or immunological systemic therapies,^23–25^ the data from this national surgical cohort support consideration of resection as one option for patients with good performance status and no clinical signs of lymph node metastasis. For patients with a higher number of lesions, and with larger lesions, the outcomes after resection, such as the high rate of recurrence within 6 months, support an individualized approach with consideration of other primary therapies than resection surgery, taking both tumor burden and tumor biology into account.^17,26^ Further studies of oncological treatment, including prospective studies and clinical trials, are needed to guide therapeutic strategies for patients with locally advanced iCCA.^7,17^

Important strengths of this study include the analysis of prospectively registered population-level data with a high coverage rate. Furthermore, data were validated at each regional center, including postoperative diagnosis, treatment details, and outcomes. Data on vital status were complete, through linkage with the National Population Registry. The multiple iCCA group consisted of patients diagnosed preoperatively with multiple lesions to exclude patients with histopathologically diagnosed microscopic satellites, where prognosis could be different from macroscopic tumor multiplicity. Limitations to the study include the lack of data on tumor localization or distribution, such as for uni- or bilateral disease or for tumor multiplicity or tumor satellites. With regard to the distinction between multiple separate tumors and tumor satellites,^2,26^ recent data including multifocal tumor genomic sequencing suggests that both distribution patterns represent locoregional clonal metastatic seeding.^27^ Together with previous sequencing investigations,^28,29^ such data also support metastatic clonal spread from one primary lesion—rather than synchronous multicentric de novo occurrence—as the main biological mechanism in multiple iCCA.^27^ A further limitation to the present study was the lack of details on the use of staged procedures and preoperative future liver remnant volume modulation. Recurrence data, even if included in the validation data collection, were limited to data reported at the regional center level, resulting in loss to follow-up. For the selected comparison cohort of liver-only localized iCCA and multidisciplinary tumor board recommendation of nonsurgical therapy, the extracted cohort was limited in extent to registrations with TNM 8th edition codes. Lastly, without granular oncological data, this analysis did not allow any direct comparison with specific oncological therapies/regimens, locoregional or systemic.

In conclusion, resection for multiple iCCA was not associated with survival benefit compared with exploration for patients with four or more lesions. With selection to surgery for patients with good performance status, smaller tumors and no signs of lymph node metastasis, resection for a limited number of lesions (two or three) yielded a median OS above 2 years. The elevated risk of recurrence after resection for multiple iCCA motivates further study of targeted and neoadjuvant oncological approaches.

## Supplementary Information

Below is the link to the electronic supplementary material.

Supplementary file1 (DOCX 213 KB)

## Data Availability

The data supporting the conclusions of this study are available from the corresponding author on reasonable request.
